# Assessing the influence of environmental gradients on grassland aboveground biomass density estimation using GEDI and multi-source remote sensing

**DOI:** 10.1038/s41598-026-51887-z

**Published:** 2026-05-12

**Authors:** Erzheng Liang

**Affiliations:** Department of Geography, The University of Hong Kong, Hong Kong, 999077 China

**Keywords:** Grassland aboveground biomass density(AGBD), GEDI L4A, Sentinel-1/2, LightGBM, Environmental gradients, Climate sciences, Ecology, Ecology, Environmental sciences

## Abstract

Grassland aboveground biomass density (AGBD) is a key indicator for assessing grassland carbon sinks and ecosystem functioning. With the rapid expansion of satellite observations, remote sensing has been widely applied to estimate grassland AGBD. AGBD is highly sensitive to environmental gradients such as precipitation, temperature, and topography; however, this contextual dependence remains insufficiently assessed in remote-sensing estimates. Focusing on grasslands in China’s Ili River Basin, this study uses GEDI L4A footprint-level AGBD as the response variable and integrates multi-source predictors from Sentinel-2 optical data, Sentinel-1 SAR, GLO-30 topography, and TerraClimate. A LASSO-screened, LightGBM-based model for AGBD retrieval was developed, and its robustness and feature mechanisms were evaluated across elevation, slope, precipitation, and temperature gradients. Results show an overall accuracy of $R^2=0.445$, RMSE = 54.62 Mg/ha, and MAE = 27.90 Mg/ha. Along topographic gradients, the model fits best at elevations of 2000–2500 m ($R^2=0.543$) and on slopes of 0–10$^{\circ }$ ($R^2=0.529$); along climatic gradients, performance is higher where annual precipitation <300 mm ($R^2=0.476$) and is optimal at mean annual temperatures of 0–5 $^{\circ }$C ($R^2=0.523$). SHAP interpretations indicate that optical reflectance and textures dominate in low-elevation, gentle-slope areas; where terrain is complex or precipitation is higher, the importance of optical textures and radar features increases; above 3000 m, the contribution of optical features declines markedly while texture/topography/radar contributions rise. This study provides a basis for context-aware AGBD mapping in the Ili River Basin and similar regions.

## Introduction

Grassland ecosystems are one of the most widely distributed terrestrial ecosystem types, covering about 40% of the global land area^1,2^. They play an irreplaceable role in maintaining ecological security, regulating regional climate, promoting carbon cycling, and supporting livestock production^3–6^. Aboveground biomass density (AGBD) in grasslands is a key indicator reflecting productivity and carbon storage, as well as a core parameter for assessing the structure and function of grassland ecosystems^7,8^. Accurate estimation of grassland AGBD is crucial for understanding carbon cycling processes and achieving sustainable utilization of grassland resources^9–11^.

Traditional grassland AGBD estimation mainly relies on field surveys using the harvesting method, which involves plot delineation, sample cutting, fresh weight measurement, drying, and weighing^12,13^. This method can achieve high accuracy but is limited by small sample sizes, time-consuming processes, and destructiveness, and it is also constrained by terrain and environmental factors^14,15^. Due to the vast area of grasslands and significant spatiotemporal variability, traditional methods are difficult to meet the monitoring needs of large-scale, long-term data^16^.

Remote sensing data provides broad spatial coverage, high spatiotemporal resolution, and excellent consistency and stability, effectively overcoming the limitations of ground measurements and offering a possibility for high-precision biomass estimation over large areas^17,18^. Optical remote sensing can characterize vegetation characteristics through spectral responses, and with the wide coverage and short revisit periods of Sentinel-2, Landsat series, and MODIS, they have become primary data sources for estimating aboveground biomass^19,20^. SAR data can acquire vegetation structural information more effectively through backscatter mechanisms associated with surface features, providing a valuable supplementary data source for biomass estimation^21^. Previous studies have shown that the VH and VV polarization backscatter from Sentinel-1 SAR data exhibit significant sensitivity to the structural properties of grasslands, enhancing their applicability in biomass assessment^22,23^. The emergence of satellite LiDAR data from GEDI has made it possible to obtain footprint-level three-dimensional vegetation structure and AGBD data on a global scale, providing a solid data foundation for constructing high-precision grassland biomass inversion models^24,25^. By combining multi-source remote sensing features with machine learning methods, spatially continuous estimation of grassland AGBD can be achieved^9,10,26,27^.

However, existing research often overlooks the impact of environmental gradients. In fact, factors such as elevation, slope, precipitation, and temperature directly determine the vegetation growth conditions and thus significantly influence the accumulation of grassland AGBD^28–30^. Precipitation can explain a large part of the AGBD variation–56% globally, 39-45% in North American grasslands, and 71-88% in Eurasian grasslands^9^. Suitable temperature conditions are the basis for grassland growth and biomass accumulation^31^. Increased temperatures may accelerate the growth of some plants or extend the growing season, thereby increasing biomass^32^. If these gradient differences are not adequately considered in model construction, it may lead to unstable performance in complex environments. Conducting grassland AGBD inversion studies across environmental gradients will not only help improve the adaptability and reliability of models but also reveal the mechanisms of multi-source features under different ecological environments.

Based on this, this study focuses on grasslands in the Ili River Basin of China, using GEDI L4A footprint-level AGBD data as the dependent variable, combined with multi-source features such as optical, radar, terrain, and climate data, to construct a LightGBM machine learning inversion model. The research objectives are: (1) to evaluate the accuracy of grassland AGBD inversion models under different environmental gradients; (2) to reveal the importance differences of multi-source features under different gradient conditions; thus providing methodological references for the precise monitoring of biomass in arid region grasslands and scientific evidence for regional grassland ecosystem management and sustainable utilization.

## Materials and methods

### Study area

**Figure 1:**
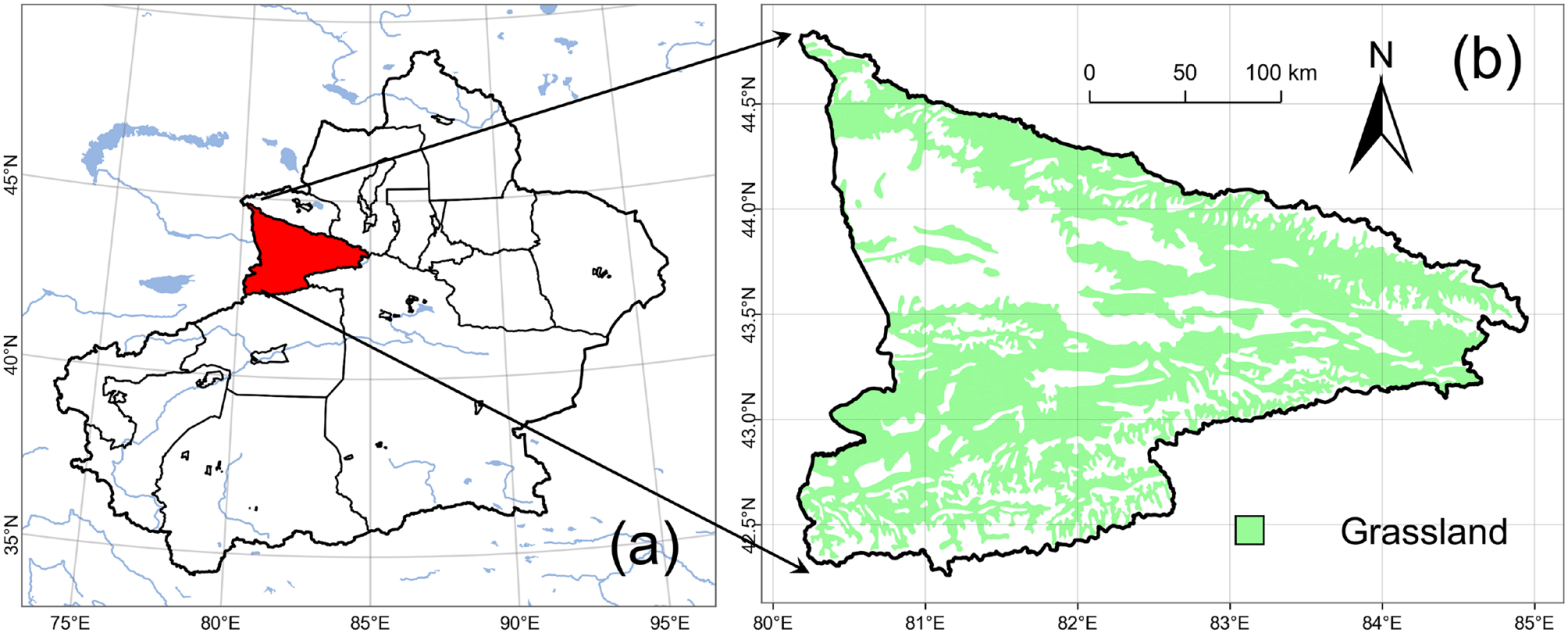
Study area map: (**a**) Location of the Ili River Basin within the Xinjiang Uygur Autonomous Region; (**b**) Grassland distribution in the Ili River Basin.

The Ili River Basin (China region) is located at the western end of the Tianshan Mountains in the Xinjiang Uygur Autonomous Region (Fig. 1), with geographical coordinates ranging from 80$^{\circ }$09’ to 84$^{\circ }$56’E and 42$^{\circ }$14’ to 44$^{\circ }$50’N. The Ili River is formed by the confluence of tributaries such as the Kash River, Gongnaisi River, and Tekes River, running from east to west through the Ili River Valley. It has a total length of about 442 km, with the basin surrounded by mountains on three sides. The terrain is shaped like a trumpet, with a higher eastern section and a lower western section, and the width of the valley increases from east to west. The total area is 57,550.80 km$^2$, with an annual runoff volume of approximately 18 billion cubic meters.

**Figure 2:**
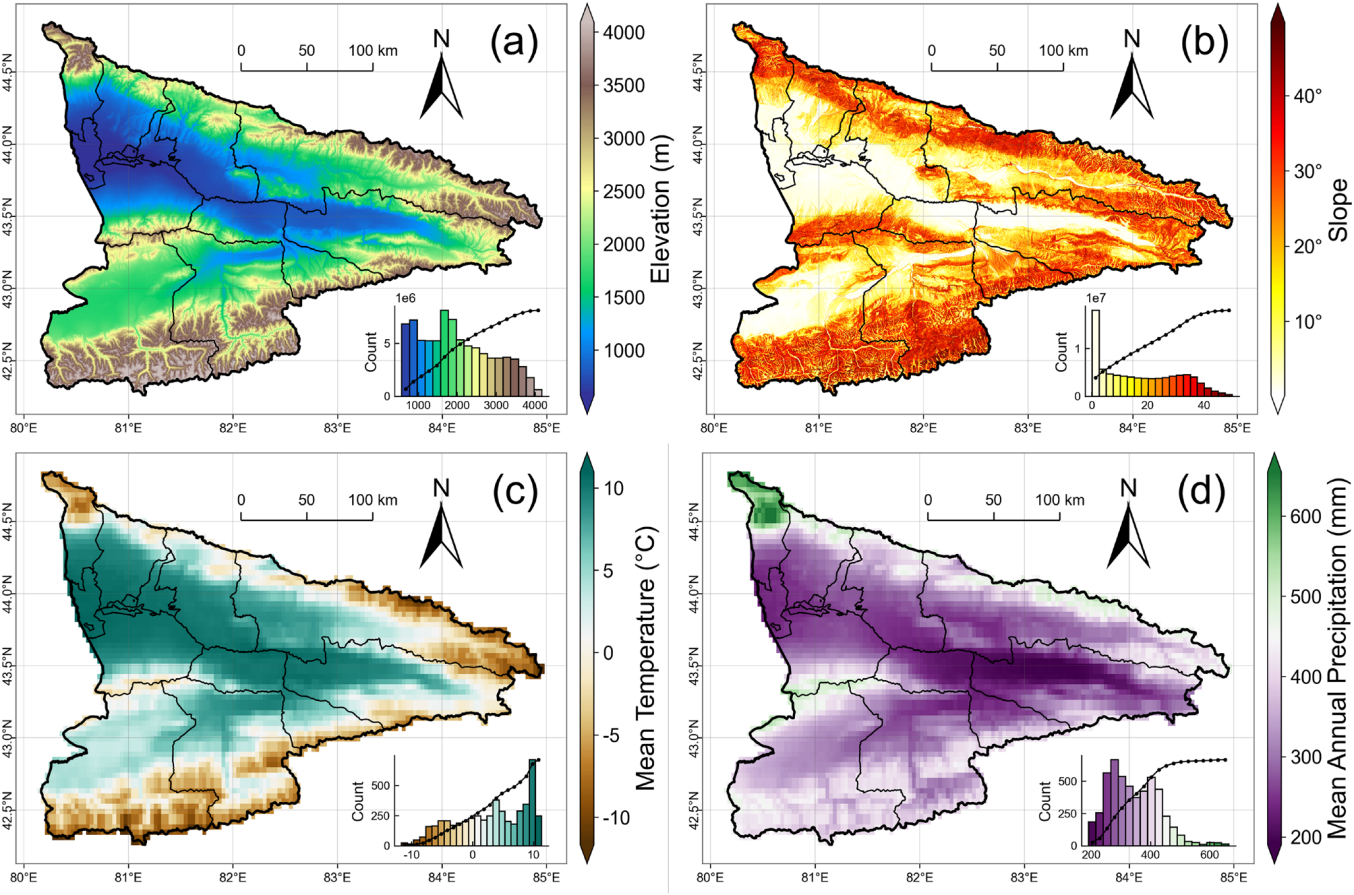
Spatial distribution of environmental gradients in the Ili River Basin: (**a**) elevation, (**b**) slope, (**c**) mean annual temperature (2003–2022), and (**d**) mean annual precipitation (2003–2022).

The natural conditions within the basin vary significantly, exhibiting distinct environmental gradients (Fig. 2; Table 1). The elevation ranges from 531 m to over 6100 m, with an average elevation of about 2011 m. The slope varies from flat terrain (0$^{\circ }$) to steep mountainous areas (83.6$^{\circ }$), with an average slope of 17.6$^{\circ }$. Precipitation is unevenly distributed across the area, with the annual average precipitation ranging from less than 200 mm to over 650 mm, with an average of about 342 mm. The temperature spans from the cold temperate zone to the temperate zone, ranging from -12.9$^{\circ }$C to 11.2$^{\circ }$C, with an average temperature of 2.9$^{\circ }$C. These differences provide a complex environmental backdrop for the distribution and growth of grassland vegetation.

**Table 1:** Descriptive statistics of ecological gradients in the Ili River Basin.

Environmental variables	Min	Max	Mean	Median	Std
Elevation (m)	531.00	6105.80	2010.87	1866.91	572.09
Slope ($^{\circ }$)	0.00	83.64	17.58	15.70	14.10
Mean annual precipitation (mm)	189.90	661.25	342.36	330.53	85.25
Mean temperature ($^{\circ }$C)	-12.96	11.19	2.93	3.68	5.83

### Data sources

#### Sentinel-2

The Sentinel-2 constellation consists of two sun-synchronous satellites, Sentinel-2A and -2B, providing 13 spectral bands that span the visible, near-infrared, and shortwave infrared, with spatial resolutions of 10–60 m and a 5-day revisit period^33,34^. In this study, we used Sentinel-2 imagery from June–September 2022 to capture multispectral information during the grassland growing season, forming the basis for constructing spectral reflectance and vegetation indices.

#### Sentinel-1

The Sentinel-1 mission comprises two C-band synthetic aperture radar (SAR) satellites, Sentinel-1A and -1B, enabling day-and-night, all-weather observations. Its primary acquisition mode is the Interferometric Wide (IW) swath, with spatial resolution up to 10 m^35,36^. We used VV- and VH-polarized backscatter data from June–September 2022 to characterize the structural attributes of grasslands.

#### GLO-30 DEM

The Copernicus Global Digital Elevation Model (GLO-30 DEM), jointly released by the European Space Agency (ESA) and the European Union, has a spatial resolution of 30 m and is among the most extensive high-accuracy public DEM products^37^. We used the GLO-30 DEM to derive topographic factors including elevation, slope, and aspect.

#### TerraClimate

The TerraClimate dataset provides monthly climate and water-balance variables since 1958, at a spatial resolution of approximately 4 km^38^. We used climatological means for 2003–2022 and 2022 anomalies of annual mean temperature, annual precipitation, VPD, and AET to characterize the climatic background of the Ili River Basin and the year-specific perturbations.

#### GEDI L4A AGBD

**Figure 3:**
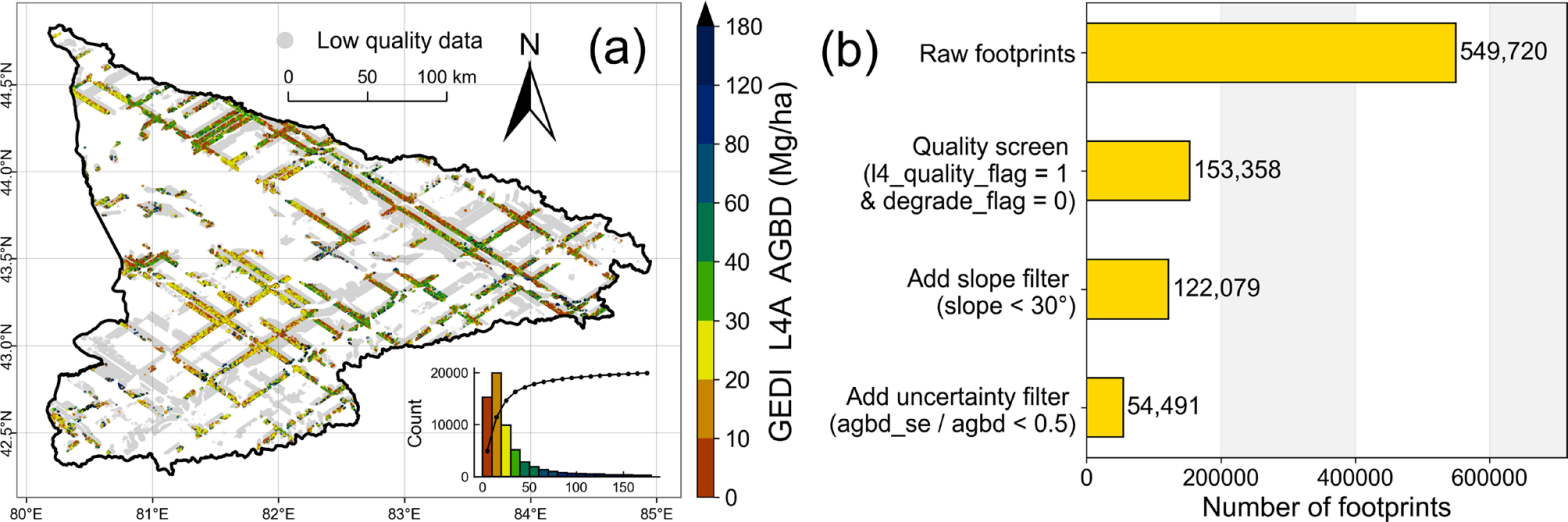
GEDI L4A footprint distribution and filtering procedure in the Ili River Basin: (**a**) spatial distribution of AGBD footprints with low-quality data in grey; (**b**) number of footprints retained after each screening step.

The Global Ecosystem Dynamics Investigation (GEDI) is the first spaceborne full-waveform LiDAR mission mounted on the International Space Station, operating since December 2018^24^. Its L4A product provides footprint-scale (25 m diameter) estimates of aboveground biomass density (AGBD) derived via parametric modeling with error control, covering global regions within the ISS ground track (±51.6$^{\circ }$ latitude)^25,39^. In this study, we assembled GEDI L4A data from June–September 2022 (the grassland growing season) and used quality-control flags to retain high-quality footprint samples for analysis (Fig. 3).

All predictor layers were sampled to a 30 m grid to form a consistent modeling dataset.

### Methods

#### Data preprocessing

To ensure spatial and temporal consistency across multi-source datasets, we first performed preprocessing. Sentinel-2 imagery was cloud-masked using the S2Cloudless approach and median-composited over the grassland growing season (June–September 2022). Sentinel-1 data were likewise median-composited over the same period to mitigate noise and orbit-related effects. GEDI L4A AGBD data were restricted to June–September 2022 and filtered using quality-control flags (l4_quality_flag=1, degrade_flag=0) together with terrain and error constraints (slope <30$^{\circ }$, agbd_se/agbd <0.5) to obtain high-quality samples. TerraClimate data were used to compute climatological means for 2003–2022 and to extract 2022 anomalies of annual mean temperature, precipitation, VPD, and AET.

#### Feature extraction

To comprehensively characterize the factors influencing grassland biomass, we constructed four categories of features: optical, radar, topographic, and climatic. The specific features are summarized in Table 2.

1. **Optical features:** From the median composite Sentinel-2 imagery, we extracted apparent reflectance for 12 bands (B2–B8, B8A, B11–B12) and computed 11 classical vegetation indices (e.g., EVI, SAVI, ARVI). In addition, eight NDVI variants (NDVI84–NDVI8A7) based on red-edge bands were constructed to enhance sensitivity to canopy physiological status.
2. **Optical texture:** Using the 8 NDVI images, we applied the gray-level co-occurrence matrix (GLCM) to compute three texture metrics–entropy, contrast, and variance–yielding 24 features in total.
3. **Radar features:** VV- and VH-polarized backscatter coefficients from Sentinel-1.
4. **Radar texture:** For VV and VH separately, we derived entropy, contrast, and variance, resulting in 6 texture features.
5. **Topographic factors:** Elevation, slope, and aspect derived from the GLO-30 DEM.
6. **Climatic factors:** Both climatology (2003–2022) and 2022 anomalies, comprising 8 variables: annual mean temperature, annual precipitation, VPD, and AET.

**Table 2:** Summary of driving variables used for LightGBM-based grassland AGBD modeling in the Ili River Basin.

Category	Variable (unit)	Description
Climatic factors (4 km)	MeanTemp_clim ($^{\circ }$C)	20-yr (2003–2022) mean annual temperature
	AnnualPrecip_clim (mm)	20-yr mean annual precipitation
	VPD_mean_clim (kPa)	20-yr mean vapor pressure deficit
	AET_sum_clim (mm)	20-yr mean annual actual evapotranspiration
	MeanTemp_anom ($^{\circ }$C)	Temperature anomaly in 2022
	AnnualPrecip_anom (mm)	Precipitation anomaly in 2022
	VPD_mean_anom (kPa)	VPD anomaly in 2022
	AET_sum_anom (mm)	AET anomaly in 2022
Topographical factors (30 m)	Elevation (m)	Derived from GLO-30 DEM
	Slope ($^{\circ }$)	Derived from GLO-30 DEM
	Aspect ($^{\circ }$)	Derived from GLO-30 DEM
Radar features (10 m)	VV, VH (dB)	Sentinel-1 C-band backscatter coefficients
	VV_contrast/ent/var	GLCM texture metrics from VV band
	VH_contrast/ent/var	GLCM texture metrics from VH band
Optical features (10–20 m)	B2–B8, B8A, B11–B12	Sentinel-2 reflectance bands (visible, NIR, SWIR)
	NDVI variants (NDVI84–NDVI8A7)	Red-edge based normalized difference indices
	EVI, SAVI, ARVI	Classical vegetation indices
	RDVI, TDVI, NBDI	Vegetation/water indices
	MNDWI	Water index
	NDVI texture: contrast/ent/var	GLCM texture metrics from NDVI variants

#### Sample grouping and dataset preparation

After screening, a total of 54,491 GEDI footprint samples were retained. To assess the influence of environmental conditions on model performance, samples were grouped by elevation, slope, long-term mean annual precipitation (AnnualPrecip_clim), and long-term mean temperature (MeanTemp_clim) (Table 3). Climate-based groupings were derived from 2003–2022 climatological averages to reflect the stable environmental background of the study area. Year-specific climate anomalies (e.g., 2022 temperature and precipitation anomalies) were used only as modeling features and not for grouping. For model development, samples within each group were randomly split into a training set (80%) and a test set (20%) to ensure independence in model evaluation.

**Table 3:** Environmental gradient groupings used for grassland AGBD modeling in the Ili River Basin.

Gradient type	Grouping scheme	Sample size (N)
Elevation (m)	500–1000	1818
	1000–1500	11382
	1500–2000	18973
	2000–2500	10017
	2500–3000	8390
	>3000	3911
Slope ($^{\circ }$)	0–10	5180
	10–20	19450
	20–30	29861
Annual precipitation (mm)	<300	17598
	300–400	26884
	>400	10009
Mean temperature ($^{\circ }$C)	<0	12108
	0–5	17600
	>5	24783

#### Model construction with LightGBM and LASSO

To retrieve grassland AGBD, we adopted the lightweight gradient boosting framework LightGBM (Light Gradient Boosting Machine). LightGBM is an ensemble learning method based on gradient-boosted decision trees that maintains high computational efficiency and accuracy with large-scale data and high-dimensional features^40^. Prior to modeling, we applied the LASSO algorithm to screen candidate variables, reducing redundancy and multicollinearity^41^. The selected features were then used as inputs to train and predict with the LightGBM model.

#### Model evaluation

To comprehensively assess predictive performance, we used three metrics: the coefficient of determination ($R^2$), root mean squared error (RMSE), and mean absolute error (MAE). $R^2$ measures goodness of fit; RMSE is more sensitive to large residuals and thus reflects errors for high-valued samples; MAE intuitively characterizes overall bias. The formulas are:

1 $$\begin{aligned} R^2 = 1 - \frac{\sum _{i=1}^n (y_i - \hat{y}_i)^2}{\sum _{i=1}^n (y_i - \bar{y})^2} \end{aligned}$$

2 $$\begin{aligned} RMSE = \sqrt{\frac{1}{n} \sum _{i=1}^n (y_i - \hat{y}_i)^2} \end{aligned}$$

3 $$\begin{aligned} MAE = \frac{1}{n} \sum _{i=1}^n |y_i - \hat{y}_i| \end{aligned}$$

where $y_i$ is the true value of the *i*-th sample (units: Mg/ha), $\hat{y}_i$ is the model prediction for that sample, $\bar{y}=\frac{1}{n}\sum _{i=1}^n y_i$ is the mean of the true values, and *n* is the number of samples used for evaluation.

#### Feature importance interpretation

To reveal the contributions of input variables to grassland AGBD estimation, we interpreted the LightGBM model using SHAP (SHapley Additive exPlanations). SHAP is based on the game-theoretic concept of Shapley values and decomposes predictions into the marginal contributions of each feature. In practice, we computed SHAP values using the trained LightGBM model and the test set, and then averaged the absolute SHAP values for each feature to obtain a global importance ranking^42^. Unlike traditional importance measures based on split counts or gain, SHAP more objectively reflects each feature’s actual contribution and mitigates biases introduced by model structure^43^.

## Results

### Overall accuracy of the grassland AGBD estimation model

**Figure 4:**
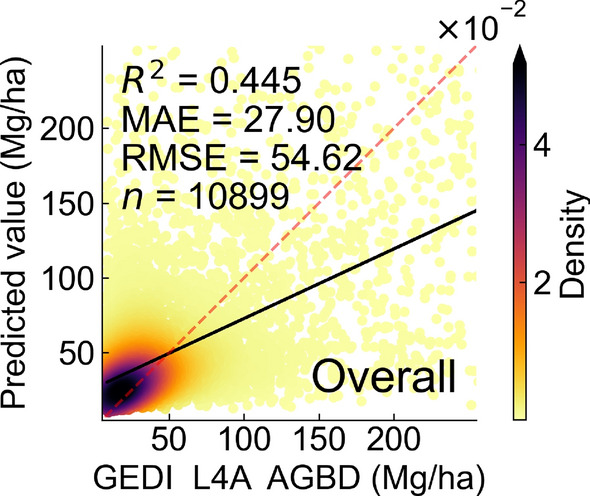
Overall performance of the LightGBM-based grassland AGBD estimation model in the Ili River Basin.

The grassland AGBD estimation model, constructed with LightGBM and LASSO-based feature selection, achieved moderate accuracy across the grasslands of the Ili River Basin. As shown in the Fig. 4, The model attained a coefficient of determination of $R^{2}=0.445$, a mean absolute error (MAE) of 27.90 Mg/ha, and a root mean squared error (RMSE) of 54.62 Mg/ha.

### Model performance across environmental gradients

#### Model performance across elevation gradients

**Figure 5:**
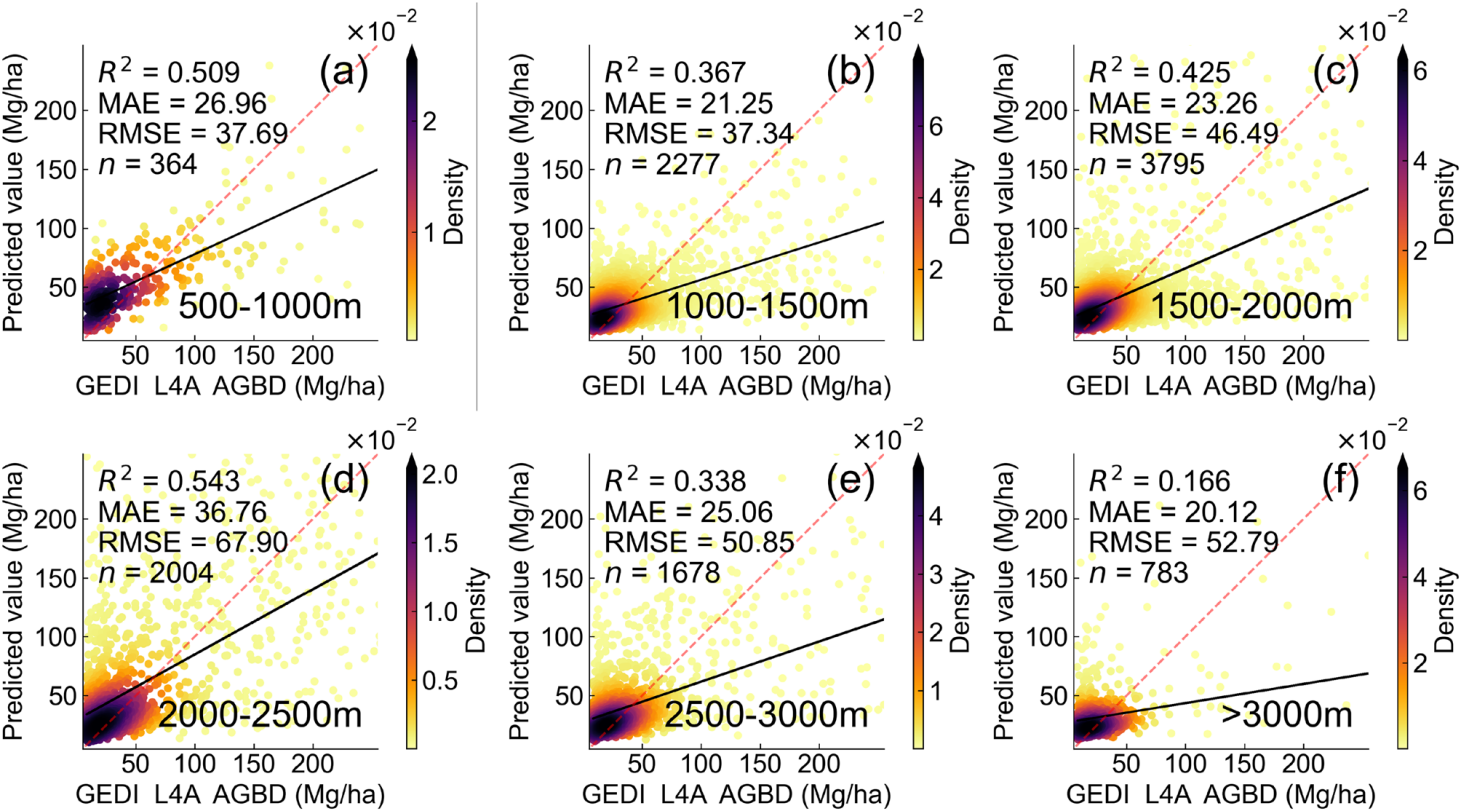
Model performance of grassland AGBD estimation across elevation gradients.

As shown in the Fig. 5, model performance varies markedly across elevation groups. In the 500–1000 m bin, the fit is better, with $R^{2}=0.509$, RMSE of 37.69 Mg/ha, and MAE of 26.96 Mg/ha ($n=364$). In the 1000–1500 m bin, $R^{2}$ decreases to 0.367 while errors remain low (RMSE = 37.34 Mg/ha, MAE = 21.25 Mg/ha, $n=2277$). For 1500–2000 m, accuracy improves ($R^{2}=0.425$) but RMSE rises to 46.49 Mg/ha ($n=3795$). The 2000–2500 m bin yields the best fit ($R^{2}=0.543$), yet errors increase substantially (RMSE = 67.90 Mg/ha, MAE = 36.76 Mg/ha, $n=2004$). In 2500–3000 m, accuracy declines again ($R^{2}=0.338$, RMSE = 50.85 Mg/ha, $n=1678$). Above $>3000$ m, the model’s fitting ability weakens notably, with $R^{2}=0.166$; although MAE is relatively low (20.12 Mg/ha), RMSE remains large (52.79 Mg/ha, $n=783$).

Overall, model accuracy tends to decrease with increasing elevation, with the poorest fit above $>3000$ m. Regarding error metrics, MAE is relatively stable across groups (approximately 20–37 Mg/ha), whereas RMSE–being more sensitive to a small number of large deviations–shows pronounced increases in the 1500–2500 m and high-elevation bins. In addition, the fitted-line slopes across groups are generally less than 1, indicating systematic underestimation at high AGBD values; this underestimation is more pronounced at higher elevations.

#### Model performance across slope gradients

**Figure 6:**
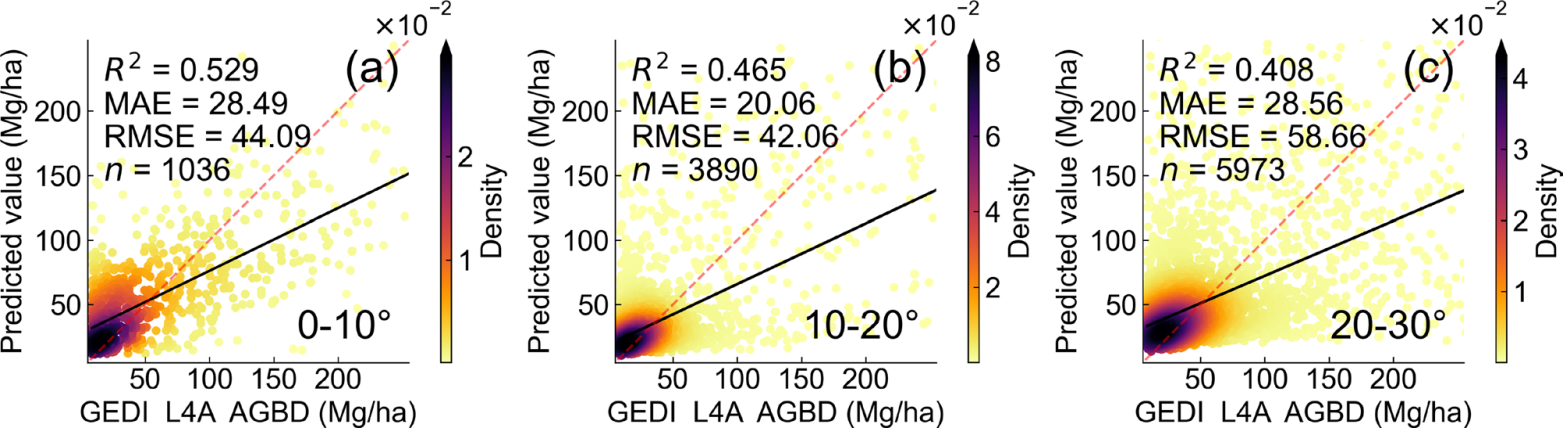
Model performance of grassland AGBD estimation across slope gradients.

Across slope-based groups, the grassland AGBD estimation model likewise exhibits varying performance. As shown in Fig. 6, in the 0–10$^{\circ }$ bin, accuracy is highest, with $R^{2}=0.529$, MAE of 28.49 Mg/ha, and RMSE of 44.09 Mg/ha ($n=1036$). In the 10–20$^{\circ }$ bin, $R^{2}$ decreases to 0.465, but errors remain lower (MAE = 20.06 Mg/ha, RMSE = 42.06 Mg/ha, $n=3890$). In the 20–30$^{\circ }$ bin, accuracy declines further to $R^{2}=0.408$, with MAE and RMSE of 28.56 Mg/ha and 58.66 Mg/ha, respectively ($n=5973$). Overall, the model performs relatively well at low (0–10$^{\circ }$) and moderate (10–20$^{\circ }$) slopes, while fitting ability weakens at higher slopes (20–30$^{\circ }$), accompanied by a marked increase in RMSE, indicating larger prediction errors in steep terrain.

#### Model performance across precipitation gradients

**Figure 7:**
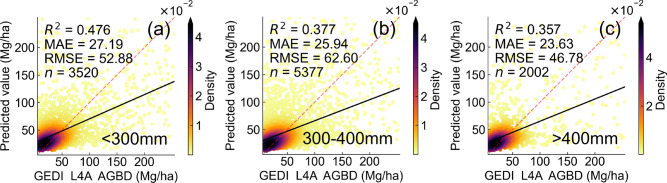
Model performance of grassland AGBD estimation across precipitation gradients.

After partitioning annual precipitation into three bins–$<300$ mm, 300–400 mm, and $>400$ mm–the model exhibits a decreasing accuracy trend with increasing precipitation. As shown in Fig. 7, in the $<300$ mm bin, accuracy is relatively high, with $R^{2}=0.476$, MAE of 27.19 Mg/ha, and RMSE of 52.88 Mg/ha ($n=3520$). In the 300–400 mm bin, the fit deteriorates ($R^{2}=0.377$), MAE is 25.94 Mg/ha, and RMSE rises markedly to 62.60 Mg/ha ($n=5377$). In the $>400$ mm bin, $R^{2}$ further decreases to 0.357, with MAE = 23.63 Mg/ha and RMSE = 46.78 Mg/ha ($n=2002$). Overall, the model performs better under low-precipitation conditions ($<300$ mm), whereas RMSE increases substantially in the mid-precipitation bin (300–400 mm), indicating larger deviations for a subset of samples. In the $>400$ mm bin, despite a lower MAE, the model fit remains weak, suggesting insufficient stability under high-precipitation conditions. Across all three scatterplots, there is pronounced underestimation at the high-AGBD end: regression-line slopes are less than 1, and this underestimation becomes more pronounced as precipitation increases.

#### Model performance across temperature gradients

**Figure 8:**
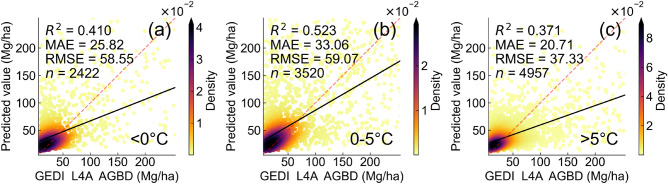
Model performance of grassland AGBD estimation across gradients.

As shown in Fig. 8, across temperature-based groups, in the $<0^\circ \textrm{C}$ bin the model attains $R^{2}=0.410$, MAE of 25.82 Mg/ha, and RMSE of 58.55 Mg/ha ($n=2422$). In the 0–5 $^\circ$C bin, the fit is highest with $R^{2}=0.523$, though errors are also relatively large (MAE = 33.06 Mg/ha, RMSE = 59.07 Mg/ha, $n=3520$). In the $>5^\circ$C bin, the model’s fitting ability declines ($R^{2}=0.371$), but errors decrease markedly (MAE = 20.71 Mg/ha, RMSE = 37.33 Mg/ha, $n=4957$). Overall, the model shows the best goodness-of-fit in the moderate-temperature range (0–5 $^\circ$C) but with higher RMSE and MAE; under warmer conditions ($>5^\circ$C), explanatory power weakens while error levels are lower, indicating a divergence between fit and error metrics across temperature gradients.

### Feature importance across environmental gradients

#### Feature importance across elevation gradients

**Figure 9:**
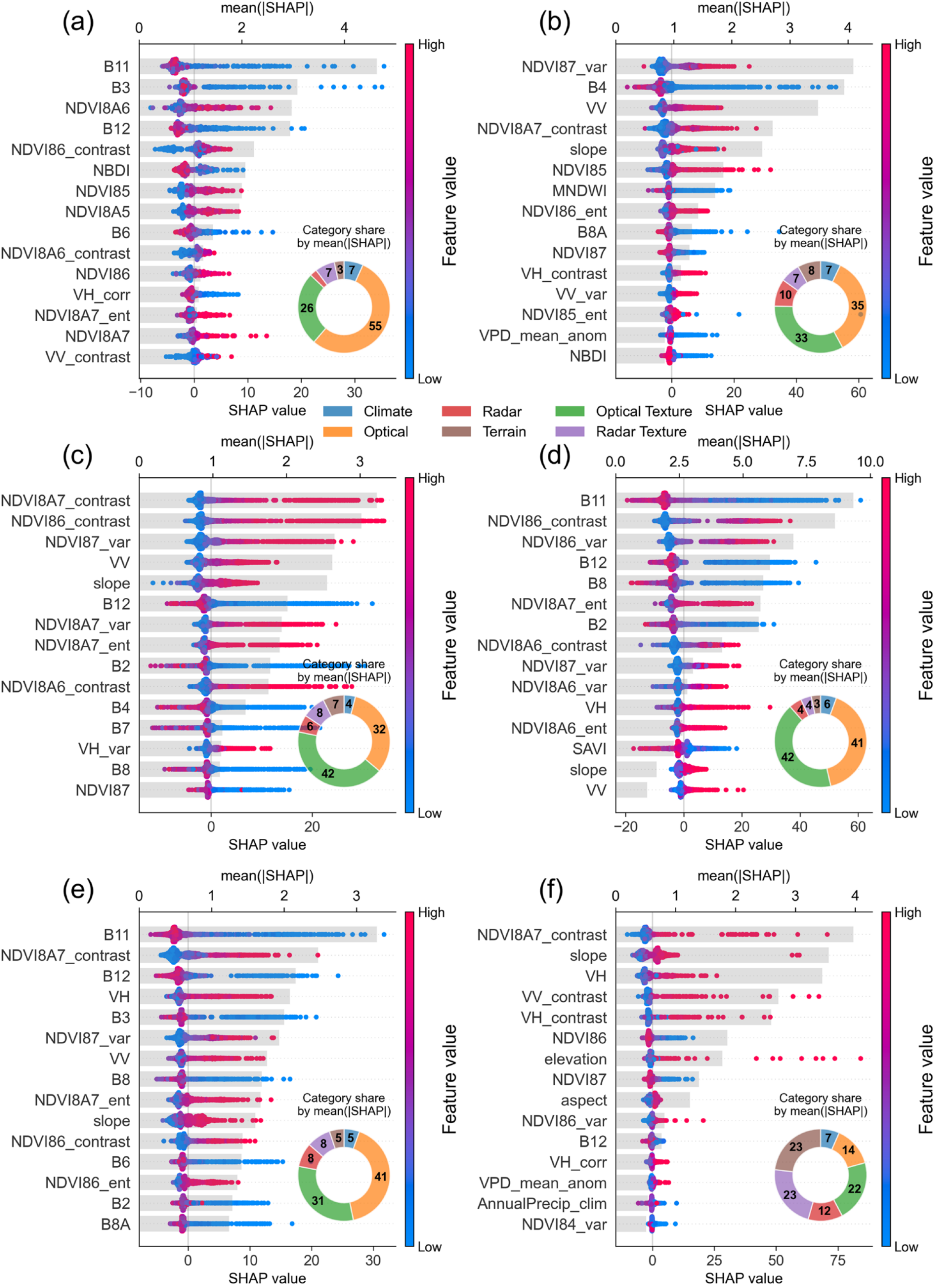
SHAP-based feature contributions for grassland AGBD models under different elevation conditions.

Across elevation bands, the ranking and category shares of feature importance differ markedly. As shown in Fig. 9, at low elevations (500–1000 m), optical bands and vegetation indices dominate the model; B11, B3, and the NDVI family rank among the top, and optical features account for more than half of the importance (54.5%), indicating that AGBD is explained primarily by spectral reflectance. As elevation rises to 1000–2000 m, the dominance of optical features diminishes, while optical texture metrics (e.g., NDVI8A7_contrast, NDVI86_contrast) move into the leading positions, with their contribution exceeding 40%. This suggests that, at mid elevations, spatial structural information of vegetation explains AGBD variation better than reflectance alone. In the 2000–3000 m band, optical and optical-texture contributions are roughly comparable; representative features include NDVI8A7_contrast, NDVI86_contrast, and shortwave-infrared bands such as B12 and B8. Meanwhile, some radar features (VV, VH and their texture metrics) begin to enter the top ten, reflecting the complementary role of radar signals at higher elevations. Above $>3000$ m, the contribution pattern shifts markedly: the share of optical features drops sharply to 14.1%, while the importance of optical texture, topography, and radar texture increases substantially (each around 20%). At this stage, features such as NDVI8A7_contrast, slope, radar polarizations, and their textures become dominant.

#### Feature importance across slope gradients

**Figure 10:**
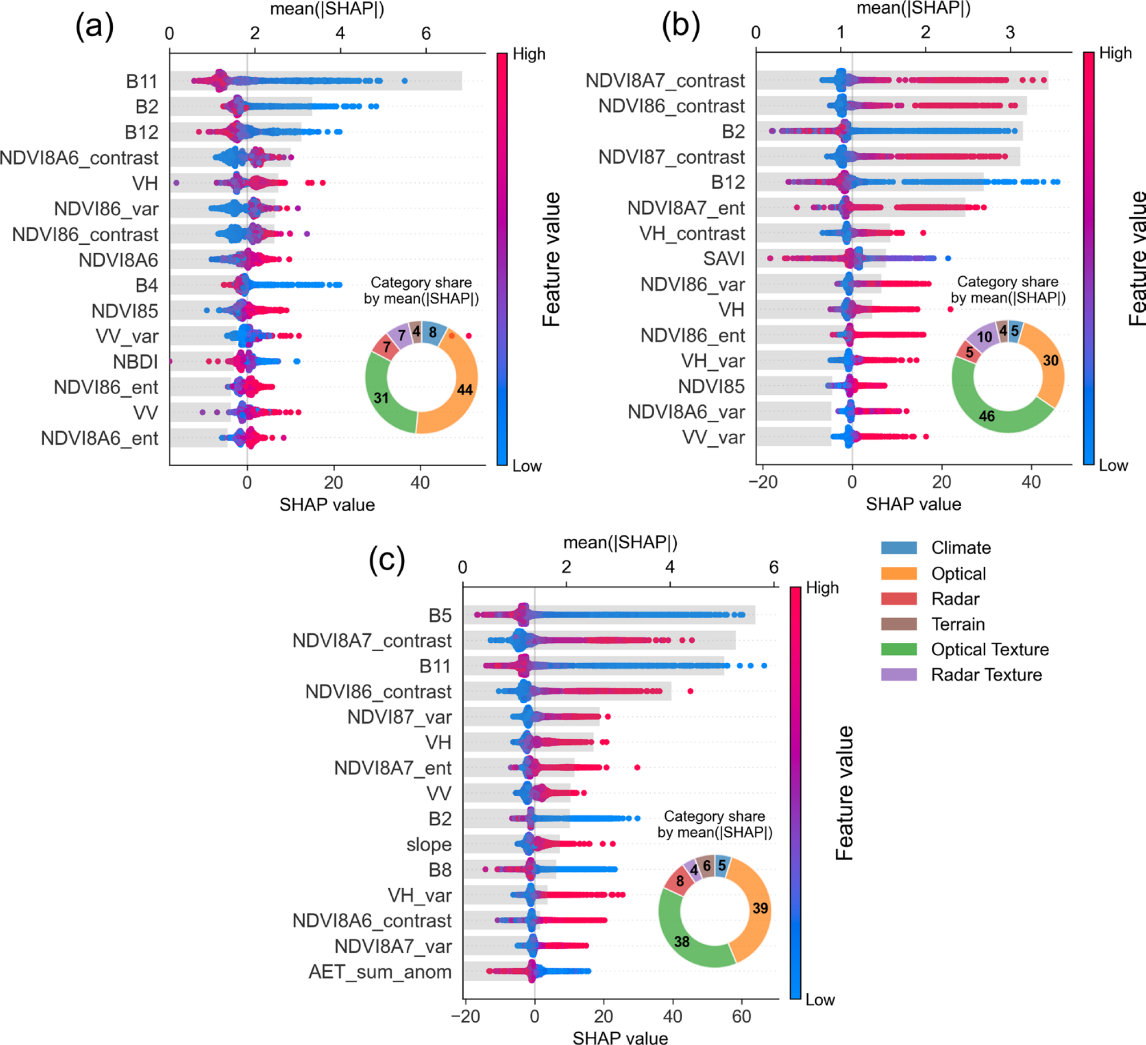
SHAP-based feature contributions for grassland AGBD models under different slope conditions.

Across slope classes, the importance pattern is broadly consistent. As shown in Fig. 10, at low slopes (0–10$^{\circ }$), optical features contribute the most (43.9%), with representative variables including B11, B2, B12, as well as several NDVI indices and texture variables. Optical texture also contributes considerably (31.0%); NDVI86_contrast, NDVI86_var, and NDVI8A6_contrast rank prominently, indicating a dual reliance on optical reflectance and texture in low-slope areas. At moderate slopes (10–20$^{\circ }$), the contribution of optical features decreases to 30.0%, while optical texture rises sharply to 46.4%, becoming the leading category. The top ten features are dominated by NDVI texture variables (e.g., NDVI8A7_contrast, NDVI86_contrast, NDVI87_contrast), suggesting that increasing slope amplifies the role of vegetation spatial structure in explaining AGBD. At higher slopes (20–30$^{\circ }$), optical features and optical texture are nearly balanced (38.9% vs. 38.1%), and radar features increase (8.5%). The leading features remain NDVI textures, but optical bands (e.g., B2, B4) and radar signals (VV, VH) also start to play a role. This indicates that in more rugged terrain, the model increasingly relies on the combined effects of multi-source features. Overall, models in low-slope areas depend mainly on optical bands with some texture information; in moderate-slope areas, optical texture dominates; and at higher slopes, optical and texture features are balanced, with radar contributions becoming more pronounced.

#### Feature importance across precipitation gradients

**Figure 11:**
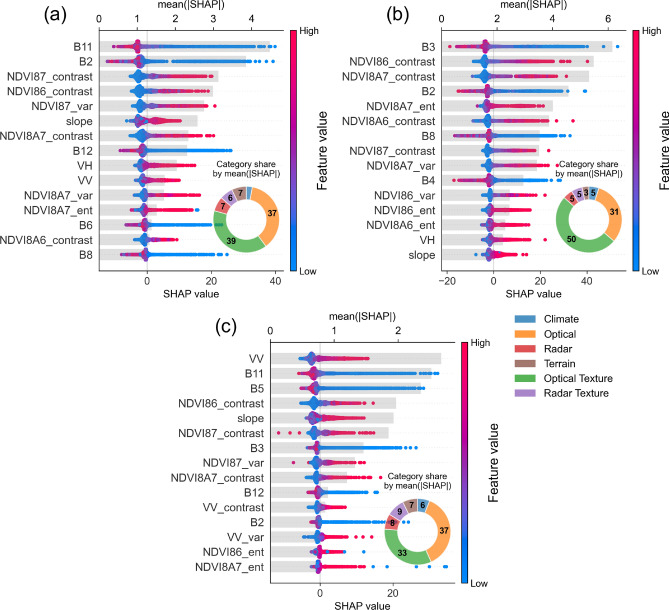
SHAP-based feature contributions for grassland AGBD models under different precipitation conditions.

Across precipitation bands, key features and category contributions differ. As shown in Fig. 11, in the $<300$ mm bin, optical bands (B11, B2, B12) and vegetation-index textures (NDVI87_contrast, NDVI86_contrast, NDVI87_var) rank at the top; optical and optical-texture categories account for 36.9% and 39.4%, respectively, jointly dominating model explanatory power. In the 300–400 mm bin, optical texture importance strengthens further (50.0%); features such as NDVI86_contrast, NDVI8A7_contrast, and NDVI87_contrast nearly occupy the entire top ten, while the contribution of optical bands weakens (31.0%). This indicates that, under moderate precipitation, vegetation spatial structure becomes the core explanatory source. In the $>400$ mm bin, the feature composition adjusts noticeably. Radar features (VV, VH) and their textures (VV_contrast, VH_var) move into the leading ranks, contributing about 18% in total–clearly higher than in low-precipitation conditions. Optical bands (B11, B5, B3) and textures (NDVI86_contrast, NDVI87_contrast) still dominate, but the category distribution is more balanced: optical features 36.7%, optical texture 33.1%, and radar features plus textures 16.6% combined. In summary, the model relies mainly on optical bands and textures under low precipitation; texture features dominate under moderate precipitation; and radar features gain influence under high precipitation.

#### Feature importance across temperature gradients

**Figure 12:**
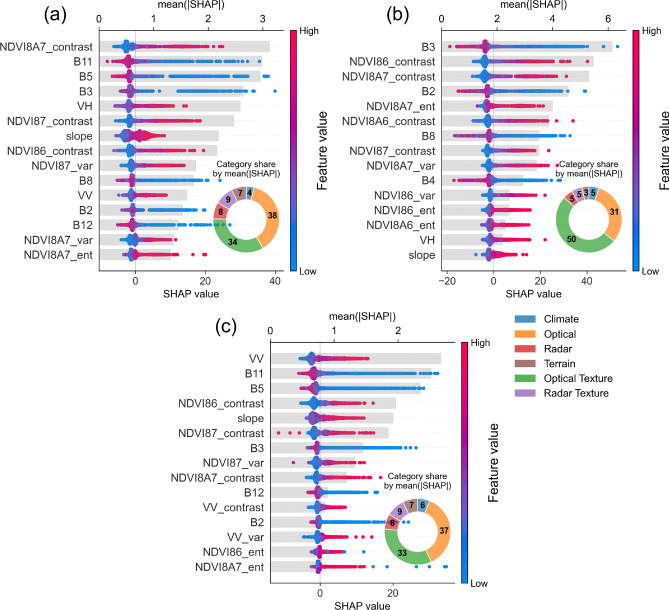
SHAP-based feature contributions for grassland AGBD models under different temperature conditions.

Across temperature bands, both the ranking of key features and the category shares vary. As shown in Fig. 12, in the $<0~^\circ$C bin, optical and optical texture jointly dominate; B11, B5, B3, and NDVI8A7_contrast, NDVI86_contrast rank highly, with optical and texture accounting for 37.8% and 33.8%, respectively–indicating that, in cold environments, the model depends on a combination of optical reflectance and vegetation structural information. In the 0–5 $^\circ$C bin, optical-texture features contribute the most (50.3%), with NDVI86_contrast and NDVI8A7_contrast consistently occupying top positions, while the role of optical bands is relatively weaker (31.4%). This suggests that, at moderate temperatures, texture differences are the primary explanatory source. In the $>5~^\circ$C bin, the model composition becomes more diverse: optical features (37.3%) and optical texture (32.9%) remain the mainstays, but the contribution of radar features and textures rises markedly (about 16% combined). VV, VH, and their texture metrics enter the top ten alongside shortwave-infrared bands such as B11 and B5. This indicates that, in warmer environments, radar signals begin to play a more prominent role. Overall, the model relies on optical and texture features in cold conditions, is dominated by texture in moderate temperatures, and, in warm conditions, draws concurrently on optical, texture, and radar information.

## Discussion

### Effects of environmental factors on the accuracy of grassland AGBD estimation

Systematic differences in AGBD estimation accuracy across ecological gradients of elevation, slope, precipitation, and temperature were observed: with increasing elevation and slope, error amplification and underestimation at high values become more pronounced; under wetter and warmer conditions, the model tends to be more conservative with weaker explanatory power. It is important to note that our reference variable is GEDI L4A footprint-level AGBD rather than ground plots. Previous studies have shown that, in mountainous and steep-slope areas, GEDI RH heights and ground geolocation are more affected by terrain, leading to reduced accuracy of the derived AGBD; this “terrain effect” is thus compounded in model evaluation, inflating errors at higher elevations and steeper slopes (e.g., evaluations in Japan and the Alps identify slope as a primary error source^44–46^). Consistent with our results, the fit is relatively high at elevations of 2000–2500 m and slopes of 0–10$^{\circ }$, but at higher elevations and steeper slopes we observe a simultaneous rise in RMSE and regression slopes below 1, indicating systematic underestimation for high-value samples alongside more outliers–patterns that align with increased heterogeneity from complex terrain and the propagation of GEDI-related errors.

Along climate gradients, high-value underestimation is more common with increasing precipitation: (i) optical indices such as NDVI tend to saturate under high cover/high moisture, weakening their response to incremental biomass^47,48^; (ii) Sentinel-1 C-band is highly sensitive to both soil and canopy water. During wet seasons or after rainfall, “moisture signals” (soil moisture, canopy water content, and intercepted water) and “biomass/structure signals” add in the backscatter, and polarization relationships shift, making it more difficult to disentangle water and biomass using C-band alone^49,50^. Notably, although the 0–5 $^{\circ }$C temperature bin attains the best fit, its error metrics are not simultaneously minimal, suggesting a potential divergence between goodness-of-fit and error magnitude across gradients: structural differentiation may be better captured under moderate temperatures, while extreme samples still elevate RMSE. Overall, cross-gradient evaluation is necessary to avoid a single overall metric masking systematic biases under complex conditions.

### Differences in feature importance across environmental factors and their mechanisms

Our SHAP analyses reveal a clear context dependence in predictor contributions. In low-elevation areas with relatively gentle terrain, optical bands and vegetation indices provide most of the explanatory power. In contrast, in high-elevation regions and in more topographically complex landscapes, the contribution of reflectance-based optical predictors decreases substantially, while the relative importance of texture measures, terrain variables, and radar-related features increases. This shift is not simply a statistical substitution among predictors; instead, it is consistent with the combined influence of ecological constraints in high-elevation grasslands and the sensing characteristics of multi-sensor remote sensing.

(1) High-elevation constraints and diminished optical informativeness.

High-elevation grasslands are typically energy- and temperature-limited. They often exhibit a shorter growing season, a more concentrated green-up period with a later peak, and a smaller seasonal amplitude in canopy development. In addition, snow accumulation and melt processes can delay green-up and shorten the effective growing window, leading to a lower seasonal amplitude and a narrower dynamic range in optical vegetation signals like NDVI during the growing season–i.e., ecological constraints translate into weakened spectral responses . Such phenological behavior has been repeatedly documented in high-latitude grasslands, where temperature and snow regimes largely control the start or end of the growing season and the magnitude of vegetation greenness dynamics^51–53^.

Moreover, high-elevation environments tend to have more bare soil background, patchy vegetation mosaics, and terrain shadows, which enhance sub-pixel mixing and dilute the vegetation signal. Together with illumination geometry effects induced by slope and aspect, these factors can systematically distort surface reflectance and reduce the stability of relationships between raw reflectance and biomass. Reviews and case studies on topographic effects consistently show that slope/aspect and illumination conditions can alter radiometric consistency and thereby affect biophysical retrieval from optical imagery in mountainous regions^54–56^.

Under high-elevation conditions, the model shifts toward structural descriptors that are less sensitive to radiometric variability. NDVI-derived GLCM textures summarize spatial heterogeneity linked to patchy grass cover and grass–bare ground mosaics, whereas elevation and slope act as ecological surrogates for temperature regimes, radiation receipt, and topographic water redistribution. Together, these factors explain the increased importance of texture and terrain features and the reduced contribution of reflectance-based optical predictors in the highest elevation bin.

**Figure 13:**
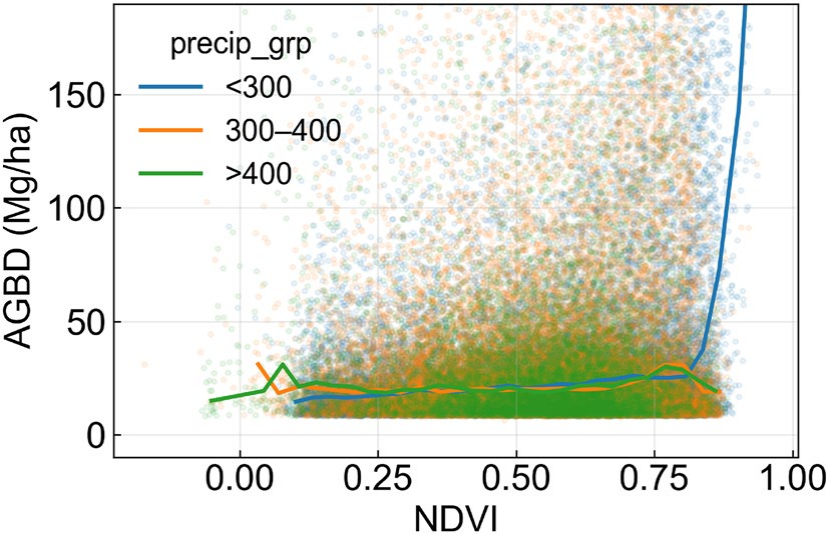
NDVI–AGBD relationships across precipitation groups.

Consistent with this interpretation, an empirical check of NDVI–AGBD relationships shows a markedly weakened monotonic association and reduced NDVI dynamic range with increasing elevation (Fig. 13, Table 4), suggesting diminished optical discriminability and increased observational instability in high-elevation terrain.

**Table 4:** Empirical relationship between NDVI and GEDI AGBD across precipitation groups.

Annual precipitation (mm)	*n*	Spearman $\rho$ (NDVI, AGBD)	NDVI range (P95–P5)
<300	17,598	0.224	0.665
300–400	26,884	0.116	0.560
>400	10,009	0.086	0.556

Spearman’s $\rho$ measures monotonic association between NDVI and AGBD. NDVI dynamic range is measured as $\textrm{P95}-\textrm{P5}$ of NDVI within each group.

(2) High-precipitation moisture effects and increased radar contribution.

Along the precipitation gradient, we observed increased importance of radar features and radar-texture metrics under wetter conditions. From an eco-physical perspective, higher precipitation is commonly associated with higher soil moisture, canopy water content, and rainfall interception. These moisture states can reduce the sensitivity of optical predictors to AGBD through at least two pathways.

First, index saturation: under higher canopy cover , NDVI and related greenness indices tends to saturate, weakening its response to incremental biomass and limiting its ability to discriminate high-biomass samples. This mechanism is consistent with our finding that underestimation at the high-AGBD end becomes more pronounced with increasing precipitation . Extensive evidence shows that NDVI saturates in high biomass, whereas indices such as EVI may maintain higher sensitivity^57^.

Second, moisture-driven spectral effects and observational instability: soil and canopy moisture can substantially alter reflectance and scattering across the VIS–NIR–SWIR domain, with SWIR being particularly sensitive to water content. As a result, “moisture variations” and “biomass variations” are more easily confounded in optical signals under wet conditions^58,59^.

In contrast, microwave SAR is strongly modulated by dielectric properties and thus responds explicitly to soil moisture, canopy water content, and rainfall interception. In C-band, near-surface soil moisture and vegetation water content jointly influence backscatter, and rainfall-related wetness can induce pronounced changes in scattering^60–62^ . The classic water cloud model (WCM) provides a theoretical framework showing how canopy water modulates backscatter via the combined effects of vegetation volume scattering and attenuation of soil returns^63^.

Therefore, in moist environments, optical reflectance and vegetation indices are often constrained by saturation and by moisture-related confounding, which reduces their effective sensitivity to biomass. In contrast, radar observables such as VV and VH backscatter, together with texture-derived measures, can better capture the coupled state of canopy structure and moisture conditions. As a result, radar features provide complementary explanatory information in machine-learning models and tend to show increased importance under wetter conditions. At the same time, the strong moisture sensitivity of C-band indicates that variations driven by moisture and those driven by biomass or structure can become more tightly entangled. This coupling may increase uncertainty and can promote systematic underestimation at the high-biomass end in wet settings. Such behavior is consistent with the weakened model fit and the amplified underestimation for high biomass observed in the high-precipitation group. From an application perspective, modeling across humid gradients can benefit from explicitly constraining the moisture background using predictors such as vapor pressure deficit and anomalies in actual evapotranspiration, which are included in our TerraClimate variables. Moreover, acknowledging moisture–biomass confounding during interpretation can improve the contextual robustness of the results.

Likewise, NDVI–AGBD curves become progressively flatter under wetter conditions and the Spearman association decreases from 0.224 (<300 mm) to 0.086 (>400 mm) (Fig. 5; Table 14), providing dataset-level evidence consistent with saturation- and moisture-related weakening of optical sensitivity.

**Figure 14:**
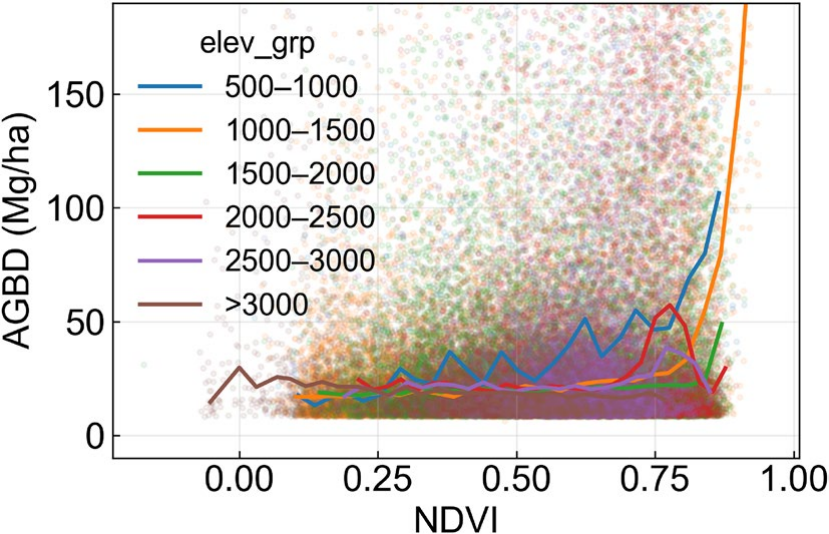
NDVI–AGBD relationships across elevation groups.

**Table 5:** Empirical relationship between NDVI and GEDI AGBD across elevation groups.

Elevation group (m)	*n*	Spearman $\rho$ (NDVI, AGBD)	NDVI range (P95–P5)
500–1000	1,818	0.417	0.690
1000–1500	11,382	0.222	0.665
1500–2000	18,973	0.081	0.546
2000–2500	10,017	0.186	0.450
2500–3000	8,390	0.122	0.368
>3000	3,911	−0.126	0.593

Spearman’s $\rho$ quantifies the monotonic association between NDVI84 and AGBD. NDVI dynamic range is measured as $\textrm{P95}-\textrm{P5}$ of NDVI within each group.

## Conclusion

This study investigated how environmental gradients regulate the performance and interpretability of satellite-based grassland AGBD mapping in the Ili River Basin. By integrating GEDI L4A AGBD with multi-source predictors (Sentinel-2 optical features, Sentinel-1 SAR metrics, topographic variables, and climate factors) and using SHAP-based interpretation, we reveal a clear context dependence in both model accuracy and feature contributions across elevation, terrain, and hydroclimatic conditions.

Model performance varies markedly along environmental gradients, with degraded skill under more extreme or heterogeneous conditions. In particular, predictions become less reliable in high-elevation and topographically complex areas and tend to exhibit stronger bias at the high-biomass end, while wetter conditions further exacerbate uncertainty and underestimation–consistent with the combined effects of ecological constraints on vegetation growth and sensor-specific observation limitations.

Feature attribution analyses indicate that the model’s “dominant information source” shifts systematically with context. In relatively low-elevation and gentle-terrain settings, reflectance-based optical predictors and vegetation indices contribute most to AGBD estimation. As elevation increases or terrain becomes more complex, the importance of optical reflectance declines and the relative contributions of texture measures, terrain proxies, and radar-related features increase, reflecting reduced optical separability and greater reliance on structural or heterogeneity cues. Under moist conditions, SAR observables and their textures become increasingly influential, plausibly due to optical saturation and moisture confounding, alongside the strong sensitivity of C-band backscatter to coupled moisture–structure states.

Several limitations should be acknowledged. First, GEDI-based reference uncertainty–especially in complex terrain–may propagate into both training and evaluation. Second, the analysis is based on a single growing season (June–September 2022), and the sensitivity of the identified gradient-dependent patterns to inter-annual variability in climate, phenology, and sensor conditions remains to be tested. Future work should therefore prioritize multi-year experiments, incorporate finer-scale hydroclimatic constraints where possible, and strengthen external validation to improve robustness in high-elevation and wet environments.

## Data Availability

The data and code that support the findings of this study are publicly available on Zenodo at https://doi.org/10.5281/zenodo.19177175.
